# CLnc-Pred: A Machine Learning Approach to Predict Long Non-Coding RNAs in Crops

**DOI:** 10.2174/0113892029416615260116163601

**Published:** 2026-03-19

**Authors:** Bhavesh Kumar Choubisa, Anu Sharma, Nitesh Kumar Sharma, Mohammad Samir Farooqi, Dwijesh Chandra Mishra, K.K. Chaturvedi, S.B. Lal, Alka Arora, Girish Kumar Jha

**Affiliations:** 1 ICAR-Indian Agricultural Statistics Research Institute, PUSA, New Delhi, 110012, India;; 2 Dr. Rajendra Prasad Central Agricultural University, Pusa, Samastipur – 848125, Bihar, India

**Keywords:** Coding RNA, lncRNA classification, machine learning, plant bioinformatics, AI, neural network

## Abstract

**Introduction:**

Long non-coding RNAs (lncRNAs) are a major class of non-coding RNAs (ncRNAs) longer than 200 nucleotides. They play key roles in plant embryogenesis, root development, reproduction, and gene silencing. Accurate identification of lncRNAs in crop species is crucial for understanding their biological functions. However, most existing computational tools are designed for human and animal lncRNAs, limiting their applicability to crop genomes. Therefore, this study aims to develop a crop-specific computational tool for the accurate classification of lncRNAs and coding RNAs in crop species, addressing the limitations of current approaches.

**Methods:**

An XGBoost classifier was trained to distinguish lncRNA and coding RNA (cRNA) sequences using sequence-intrinsic features derived from five crop species: wheat, sorghum, rice, soybean, and maize. Model performance was evaluated against benchmark tools, CPC2 and PLEKv2

**Results:**

The trained XGBoost classifier achieved an accuracy of 95.30%, precision of 93.90%, recall of 98.40%, F1-score of 96.10%, and an area under the ROC curve (AUC-ROC) of 99.40%, outperforming existing tools. These results demonstrate the model’s reliability in distinguishing lncRNAs from coding RNAs.

**Discussion:**

The trained XGBoost classifier was deployed as CLnc-Pred, a web-based application that allows users to input or upload FASTA sequences for lncRNA prediction. This framework enables efficient and accurate identification of lncRNAs in crop species.

**Conclusion:**

CLnc-Pred enhances accessibility and accuracy in crop lncRNA research and supports downstream functional and regulatory analyses. Future work will focus on expanding datasets, incorporating additional plant species, and extending the framework to support multi-class classification of diverse ncRNA types.

## 1 INTRODUCTION

Recent research highlights the importance of the transcription process in organisms. Non-coding transcripts play an important role in the transcription process of animals and plants [1]. The non-coding RNAs (ncRNAs) are a type of RNA that cannot be translated into proteins, but they play an important role in regulating the expression of coding transcripts [2]. There are various types of non-coding RNAs available in nature, of which long non-coding RNAs (lncRNAs) are one of them. The minimum length of an lncRNA transcript is 200 nt, which is a main characteristic. LncRNAs cannot be translated into proteins, similar to other non-coding RNAs. There are numerous lncRNAs identified in plants and animals, but their specific biological functions are still unexplored [3-7], especially in the field of agriculture. According to recent studies, certain newly discovered lncRNAs can regulate crucial biological processes through various mechanisms like target mimicry, histone modifications, and interactions with chromatin-modifying complexes. Therefore, to uncover their roles in various biological processes, it is very important to identify them with high accuracy.

Although numerous tools are available, such as the Coding Potential Calculator 2 (CPC2), Coding-Non-Coding Index (CNCI), Coding-Potential Assessment Tool (CPAT), lncRScan-SVM, PLEK (Predictor of Long non-coding RNAs and messenger RNAs based on k-mer schemes), PLncPRO, and PLEKv2 are available for this purpose [8-14]. The majority of these tools are trained on human or other organisms’ data and thus perform best on closely related species. However, these tools are found to exhibit poor performance on crop data, as shown in the literature [15, 16]. Thus, despite the availability of existing tools, their limited effectiveness on crop-specific data shows the need for a dedicated solution for plant transcriptomes.

Crop-specific, highly accurate methods for the prediction of lncRNAs are required. A new tool called CLnc-Pred (Crop Long non-coding RNA Prediction), which classifies transcripts into coding and long non-coding categories, has been developed using XGBoost (eXtreme Gradient Boosting) [17] to predict lncRNAs for crops. CLnc-Pred is trained on wheat, sorghum, rice, soybean, and maize lncRNA datasets.

The major contributions of this study include: (i) utilizing existing lncRNA databases for comprehensive crop-specific datasets; (ii) improving machine learning-based models for lncRNA classification; and (iii) comparing the developed model with existing tools across various crops. The accuracy of CLnc-Pred is compared against other important available tools, CPC2 and PLEKv2. CLnc-Pred performed well on the known collection of crop lncRNAs, which were taken from the PLncDB v2.0 database [18]. CLnc-Pred performs better and will serve as a valuable resource for advancing our understanding of lncRNA biology in crops.

## 2 MATERIALS AND METHODS

### 2.1 Work Flow of the Study

**Fig. (1):**
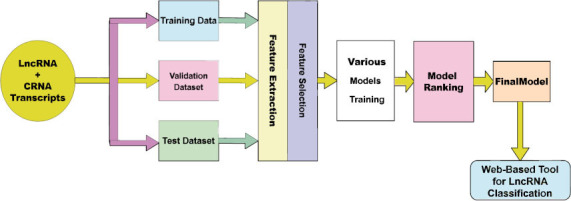
**Step-by-step workflow for developing the LncRNA classification tool.** Figure illustrating the systematic approach followed in this study, from dataset preparation and feature extraction to model training and deployment.

A dataset of transcripts was split into training, validation, and testing sets. Features were extracted and refined through feature selection, then used to train various models. The best-performing model was identified through model ranking and deployed as a web-based LncRNA classification tool (Fig. **1**).

### 2.2 Dataset Description

**Fig. (2):**
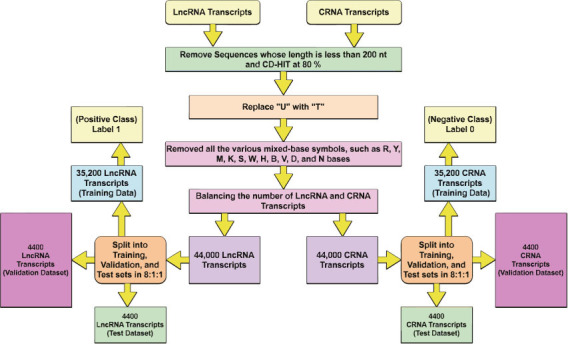
**Data pre-processing for model training.** The diagram illustrates the preprocessing and dataset preparation workflow for lncRNA and cRNA transcript classification. Sequences shorter than 200 nt and with CD-HIT similarity ≥80% were removed, ‘U’ was replaced with ‘T’, and ambiguous bases were eliminated. The dataset was balanced to 44,000 transcripts each for lncRNA (label 1) and cRNA (label 0), then split into training, validation, and test sets in an 8:1:1 ratio, with 4,400 sequences each for validation and testing per class.

We downloaded a total of 74,161 lncRNA sequences from the GREENC database [19] and 74,161 cRNA (mRNA) FASTA sequences from the EnsemblPlants database [20] for 5 crops: *Triticum aestivum* (wheat), *Sorghum bicolor* (sorghum), *Oryza sativa* (rice), *Glycine max* (soybean), and *Zea may*s (maize). Similar sequences or duplication were removed using Cluster Database at High Identity with Tolerance (CD-HIT) at 80%, 85%, 90%, 95%, and 100% [21]. An 80% identity threshold was selected to balance sequence diversity and computational efficiency while ensuring the removal of highly similar sequences. The goal is to minimize the overall size of the dataset while retaining all sequence information by deleting only redundant (or highly similar) sequences. All occurrences of ‘U’ were replaced with ‘T’, and mixed-base symbols were removed. The bias of the model towards the category with more samples is handled by randomly selecting an equal number of data points for each category (44,000 cRNA and lncRNA transcripts for each). The training, validation, and test dataset comprises 35,200, 4,400, and 4,400 sequences for each class (Fig. **2**) and Table **1**.

### 2.3 Feature Extraction

We have extracted the features such as sequence length, ORF coverage, longest ORF, GC content, normalized codon (trimer) frequency, and tetramer frequency using in-house developed Python scripts [22-25]. These features were selected based on prior knowledge about coding and non-coding transcripts [26-44]. Sequence length is the total number of nucleotides in an RNA transcript (Eq. 1). It is determined by counting how many nucleotides A, T, G, and C are present in the sequence. An ORF, or open reading frame, is a sequence of nucleotides that ends with a stop codon (TAA, TAG, or TGA) and begins with a start codon (often ATG). The longest ORF is the longest continuous section between a start and a stop codon in a sequence that constitutes a legitimate ORF (Eq. 2). ORF Coverage (Open Reading Frame Coverage) refers to the proportion of a given RNA sequence that is covered by the longest open reading frame (ORF). It is often expressed as a percentage (Eq. 3). GC content is the proportion of guanine (G) and cytosine (C) in the sequence (Eq. 4). A codon is a sequence of three nucleotides (also called a triplet) in RNA that codes for a specific amino acid or a stop signal during protein synthesis. There are (4^3^) 64 codons are possible in an RNA sequence. Codon count is the count of each codon in the sequence. We obtained the normalized codon frequencies; the count of each codon is divided by the total number of codon counts in the sequence. This normalization yields the relative frequency of each codon, and captures its proportion in the sequence (Eq. 5). Tetramer frequency is the frequency of four-letter nucleotide substrings in the sequence (Eq. 6).

Let’s the nucleotide sequence(S) with length(L) = *n*:


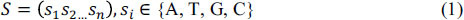


Where S is the entire nucleotide sequence.

**𝑠_𝑖_ : *i^th^*** nucleotide in the sequence.

*n:* Total number of nucleotides in the sequence.

**{A, T, G, C} =** A: Adenine, T: Thymine,

G: Guanine, C: Cytosine

Then, the extracted features are:

Longest Open Reading Frame (ORF):


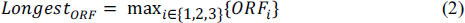


Where, *ORF*_𝑖_ is the length of the *ORF* in reading frame *i.*

ORF Coverage (%):


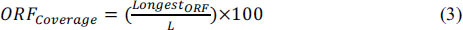


GC Content (%):


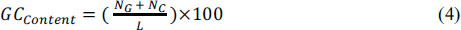


Where, N_G_ and N_C_ are the number of G and C nucleotides, respectively.

Normalized Codon (Trimer) frequency Vector (n_3_):


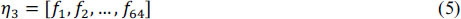


Where:


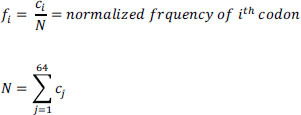


N = The total codon counts of all 64 codons


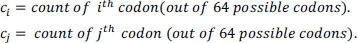


Tetramer (4-mer) Frequency Vector (φ_4_):


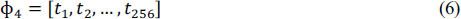


Where t_i_ is the frequency of i^th^ tetramer(4-mer) and is calculated by 
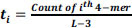
 and L - 3 is total number of possible 4-mers in the sequence of length *L (Where, L≥4).*

### 2.4 Feature Selection Using SHAP

**Fig. (3):**
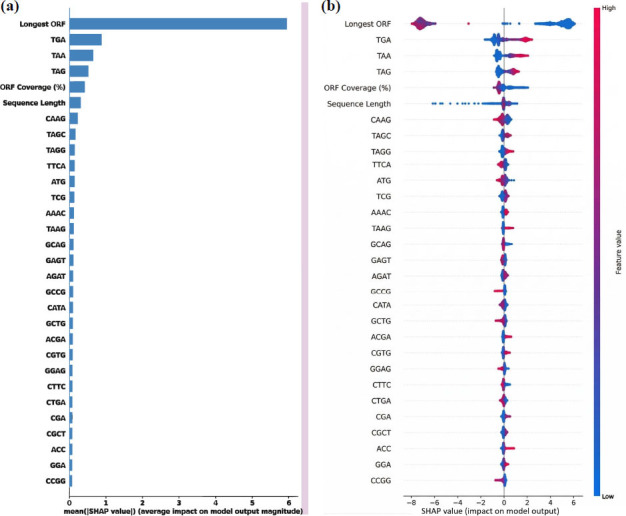
**SHAP-based feature importance analysis.** (**a**) The mean SHAP values highlighting the most influential features, with Longest ORF emerging as the top contributor, followed by stop codons (TGA, TAA, TAG), ORF Coverage (%), and Sequence Length. (**b**) The SHAP summary plot, illustrating the magnitude and direction of each feature’s impact on predictions. Together, these plots confirm the biological and statistical relevance of key sequence-derived features in distinguishing long non-coding RNAs.

In the development of predictive models for lncRNA classification, selecting the most informative features is essential for improving both model performance and interpretability. To address this, we employed SHAP (Shapley Additive exPlanations) [45], which helps in identifying the most influential sequence-derived features for predictions. By focusing on features with high SHAP values, we were able to reduce noise, eliminate redundancy, and enhance the biological relevance and transparency of our model. We selected the top 30 most important features based on SHAP values (Fig. **3**), which yielded comparable performance to the full feature set.

### 2.5 AI-based Techniques

Five models, including Convolutional Neural Networks (CNN) [46], Random Forest [47], Artificial Neural Networks (ANNs) [48], Deep Neural Networks (DNNs) [49], eXtreme Gradient Boosting (XGBoost) [17], were trained. The trained models were validated using accuracy (Eq.7), precision (Eq. 8), recall (Eq. 9), and F_1_-score (Eq. 10).


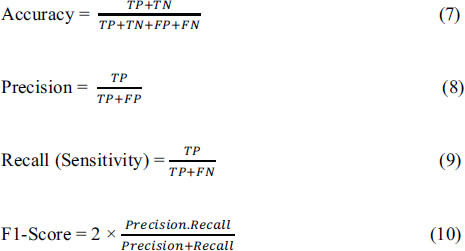


Where:

TP: Number of true positives (long non-coding transcripts correctly classified as lncRNA)

TN: Number of true negatives (coding transcripts correctly classified as cRNA)

FP: Number of false positives (coding transcripts incorrectly classified as lncRNA)

FN: Number of false negatives (lncRNA incorrectly classified as cRNA)

### 2.6 TOPSIS for Model Evaluation

Finally, we have calculated the Technique for Order Preference by Similarity to Ideal Solution Scores (TOPSIS) [50] based on 5-fold cross-validation accuracy, precision, recall, specificity, and F1-score [51]. TOPSIS was employed to rank models based on multiple evaluation metrics, allowing an objective and comprehensive selection of the best-performing model. Finally, we get the best performing model, XGBoost, among all.

### 2.7 XGBoost Objective Function

XGBoost model optimizes the following regularized objective function 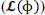 (Eq. 11):


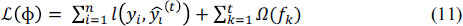


Where:


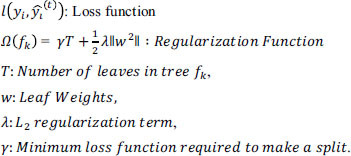


### 2.8 Hyperparameters Used in XGBoost Model

The hyperparameter set of the XGBoost model was selected using grid search and evaluated using 5-fold cross-validation [52]. The best-selected hyperparameter set is shown in Table **2**.

### 2.9 Functionality and Implementation

We have trained the models by using the Python [53] programming language, and the Google Colaboratory platform [54], which leverages the open ecosystem of Python, including abundant scientific computation and visualization libraries such as Biopython [55], NumPy [56], pandas [57], and Matplotlib [58].

## 3 RESULTS

### 3.1 Comparative Evaluation of Model Performance Using Friedman and Nemenyi Tests

**Fig. (4):**
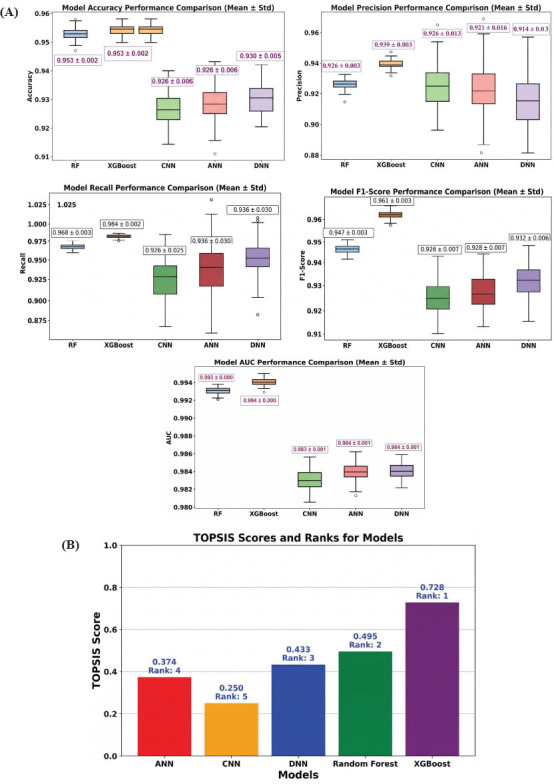
Performance Comparison of Models Across Evaluation Metrics (Mean ± SD). (**A**) Box plots of model performance on 5-Fold Cross-Validation with 6 Replications across accuracy, precision, recall, F1-score, and AUC, with XGBoost consistently leading (accuracy: 0.953 ± 0.002, precision: 0.939 ± 0.003, recall: 0.984 ± 0.002, F1-score: 0.961 ± 0.003, AUC: 0.994 ± 0.000). (**B**) shows the TOPSIS scores, where XGBoost ranks highest (0.720, rank 1), followed by Random Forest (0.495) and DNN (0.443), while ANN and CNN receive lower scores (0.374 and 0.250, respectively). Overall, both cross-validated metrics and TOPSIS confirm XGBoost as the top-performing model.

**Fig. (5):**
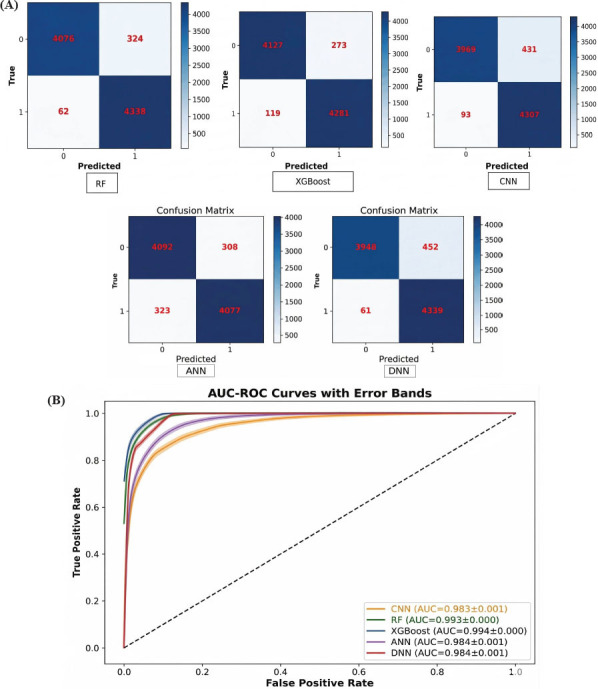
**Confusion Matrices and ROC Curves of Trained Models.** (**A**) shows the confusion matrices, while (**B**) displays the corresponding ROC curves, highlighting each model’s discriminative performance across 5-fold cross-validation with 6 replications. The ROC comparison clearly indicates that XGBoost achieves the highest AUC, demonstrating superior classification capability among all models.

We evaluated model performance using 5-fold cross-validation with 6 replicates per metric. The Friedman test showed significant differences among models (*p* < 0.0001), and post-hoc Nemenyi analysis indicated that RF and XGBoost consistently outperformed CNN, ANN, and DNN, which performed similarly (Table **S1**) [59, 60]. XGBoost achieved strong results with an accuracy of 0.953 ± 0.002, precision 0.939 ± 0.003, recall 0.968 ± 0.003, F1-score 0.953 ± 0.002, and AUC-ROC 0.994 ± 0.000 (Fig. **4A**). TOPSIS analysis further supports this finding, ranking XGBoost highest (0.720, rank 1) among all models (Fig. **4B**). The confusion matrix (Fig. **5A**) and its visualization (Fig. **5B**) confirm its robust classification ability.

### 3.2 Class-wise Classification Report for XGBoost (Mean ± SD with SE)

The XGBoost model’s class-wise performance was evaluated on cRNA (class 0) and lncRNA (class 1) using precision, recall, and F1-score (Table **S2**). For cRNA, the model achieved a precision of 0.967 ± 0.003 (SE = 0.000), recall of 0.937 ± 0.003 (SE = 0.001), and F1-score of 0.952 ± 0.002 (SE = 0.000). For lncRNA, it achieved a precision of 0.939 ± 0.003 (SE = 0.001), a recall of 0.968 ± 0.003 (SE = 0.000), and an F1-score of 0.953 ± 0.002 (SE = 0.000). These results indicate that the model predicts cRNA with high accuracy while effectively identifying lncRNA sequences. The nearly identical F1-scores for both classes highlight the model’s balanced and consistent performance, confirming its robustness and reliability in distinguishing between coding and non-coding transcripts (Fig. **S1**).

#### 3.2.1 Statistical Analysis of Class-wise Performance Metrics

We analyzed whether the XGBoost model performed differently on cRNA and lncRNA by comparing class-wise precision, recall, and F1-score (Table **S3**). Both paired t-tests and Wilcoxon signed-rank tests showed significant differences for all three metrics. Precision had *p*-values of 3.58 × 10^−27^ (t-test) and 1.86 × 10^−9^ (Wilcoxon), recall had 5.07 × 10^−28^ and 1.86 × 10^−9^, and F1-score had 2.96 × 10^−10^ and 2.35 × 10^−6^. These results suggest the model consistently distinguishes between cRNA and lncRNA, reflecting real differences in their feature patterns.

### 3.3 XGBoost Performance at Various CD-HIT Thresholds

XGBoost performance was evaluated across CD-HIT clustering thresholds of 80%, 85%, 90%, 95%, and 100% (Table **S4**). The model maintained consistently high accuracy, precision, recall, and F1-score across all thresholds, demonstrating strong robustness to changes in sequence similarity. The 80% threshold provided the best overall balance between sequence diversity and predictive performance, achieving an accuracy of 0.957 ± 0.002, precision of 0.944 ± 0.003, recall of 0.973 ± 0.002, F1-score of 0.958 ± 0.002, and AUC of 0.994 ± 0.000. These results suggest that reducing sequence redundancy enhances data diversity without compromising the model’s predictive power, confirming XGBoost’s reliability and stability across varying sequence similarity levels (Fig. **S2**).

### 3.4 XGBoost Performance with Balanced and Imbalanced Classes

The model was evaluated across different lncRNA:cRNA ratios ranging from 1:1 to 1:10 (Table **S5**). The best performance was observed with the balanced 1:1 dataset, particularly for recall and F1-score, showing that the model was highly sensitive in identifying lncRNAs. As the dataset became more imbalanced, accuracy improved slightly from 0.950 ± 0.002 (1:1) to 0.978 ± 0.002 (1:10), and precision relatively stable (~0.91–0.95). However, recall gradually dropped from 0.996 ± 0.001 to 0.804 ± 0.015, leading to a decrease in the F1-score from 0.953 ± 0.002 to 0.868 ± 0.009. Even with these changes, the AUC stayed consistently high (~0.993), indicating that the model maintained a strong ability to distinguish between classes. Overall, these findings suggest that using a balanced 1:1 dataset provides the most reliable and sensitive detection of lncRNAs (Fig. **S3**).

#### 3.4.1 Post Hoc Analysis of Model Performance on Different Dataset Ratios

We evaluated the impact of class imbalance using Kruskal–Wallis and Dunn’s tests [61]. Extreme ratios (1:1 *vs* 1:10) significantly affected performance (*p* < 0.001), while moderate ratios showed no effect (*p* > 0.05), indicating stability with balanced data. The final model was thus trained on a 1:1 lncRNA:cRNA ratio for reliable predictions (Table **S6 – S9**).

### 3.5 CLnc-Pred: A Tool for Crop LncRNA Prediction

**Fig. (6):**
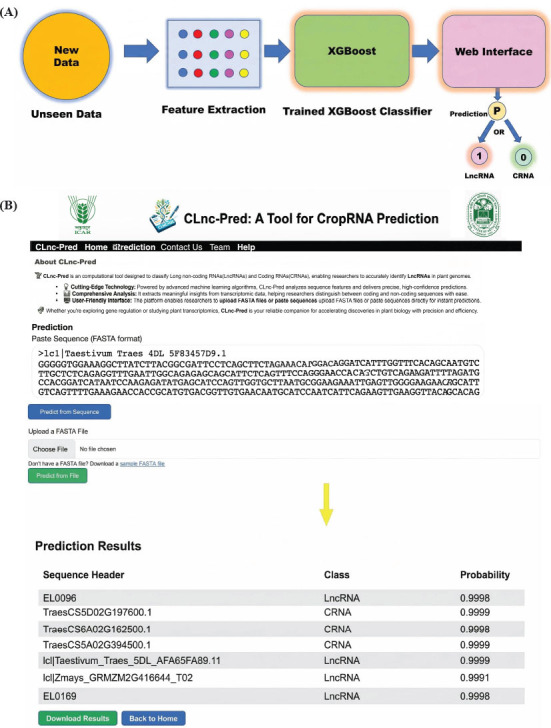
**Workflow and web interface of CLnc-Pred.** (**A**) Transcript sequences are processed to extract features such as ORF, GC content, and tetramer frequency, which are used by the XGBoost classifier to predict lncRNA (1) or cRNA (0). (**B**) The web interface allows users to upload FASTA files or paste sequences for instant predictions, displaying results with Sequence Header, Class, and Probability columns, downloadable as a CSV file.

The final model has been deployed as a web-based tool named CLnc-Pred, designed to classify unknown transcript sequences and label them as either potential long non-coding (1) or coding (0) sequences. The CLnc-Pred extract key features such as ORF metrics, GC content, and tetramer frequency from each transcript, which are then used by an XGBoost classifier to predict whether the sequence is an lncRNA or a cRNA (Fig. **6A**).

The CLnc-Pred web interface provides a user-friendly platform for researchers to upload FASTA files or paste sequences directly for instant predictions. The results are displayed in a table containing Sequence Header, Class, and Probability columns (Fig. **6B**). Users can also download the predictions as a CSV file for further analysis.

The tool is freely available at: https://clncpred.onrender.com/

### 3.6 Comparison and Evaluation of CLnc-Pred

**Fig. (7):**
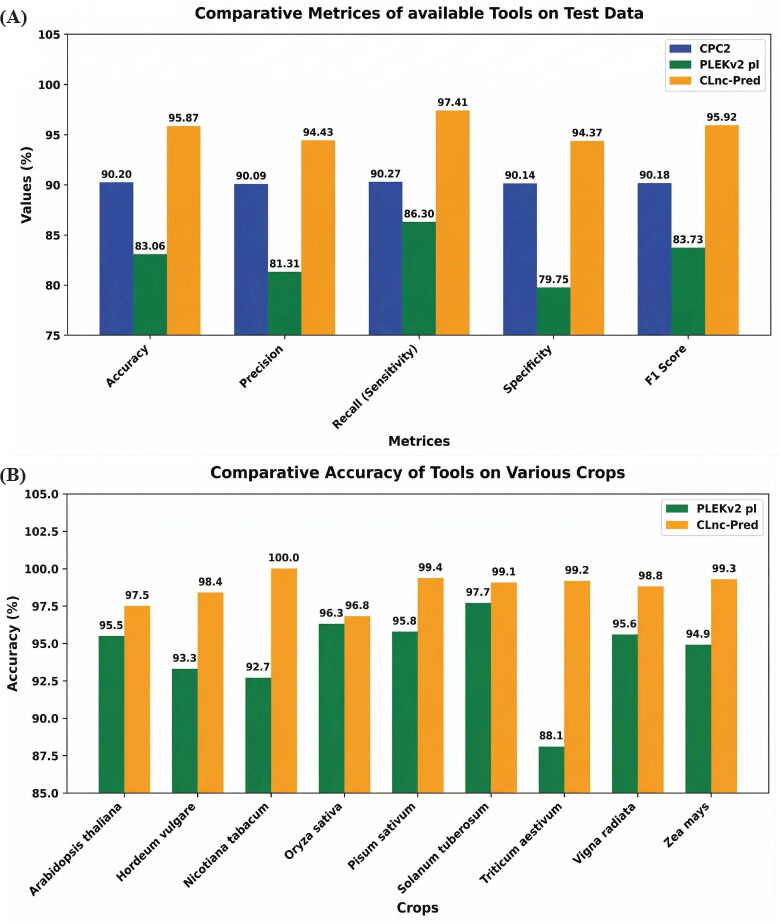
**Comparative Performance of CLnc-Pred.** CLnc-Pred consistently outperforms existing tools, including CPC2 and PLEKv2 pl, across all evaluation metrics. (**A**) It achieves the highest overall accuracy (95.87%) and F1-score (95.92%), while CPC2 shows moderate performance (~90%), and PLEKv2 pl performs lowest. (**B**) Across ten crop species, CLnc-Pred attains near-perfect accuracy (100% in Nicotiana tabacum; 99.1–99.3% in key crops), whereas PLEKv2 pl ranges from 88.1–97.7%, demonstrating CLnc-Pred’s reliability, generalizability, and effectiveness.

The CLnc-Pred was evaluated on test data and consistently outperformed existing tools, including CPC2 and PLEKv2 pl [**10**, **16**], across all metrics (Fig. **7A**, Table **S10**). When tested on well-known lncRNAs from multiple crops, *Arabidopsis thaliana*, *Hordeum vulgare*, *Nicotiana tabacum*, *Oryza sativa*, *Pisum sativum*, *Solanum tuberosum*, *Triticum aestivum*, *Vigna radiata*, and *Zea mays* (PLncDB v2.0) [18], as well as *Cyamopsis tetragonoloba* (CbLncRNAdb) [62], CLnc-Pred achieved 96.8–100% accuracy, surpassing PLEKv2 pl and demonstrating strong reliability and broad applicability across diverse crop species (Fig. **7B**).

## 4 DISCUSSION

The proposed CLnc-Pred achieved 95.30% accuracy, 93.9% precision, and 98.40% recall, indicating that the model effectively differentiates between coding and non-coding RNA sequences. Additionally, the F1-score (96.10%) confirms a strong balance between precision and recall. The AUC-ROC score of 99.40% further highlights the model’s robustness. Our approach demonstrates a significant improvement in classification performance and balances high accuracy with computational efficiency as compared to existing lncRNA prediction tools such as CPC2 and PLEKv2 pl [10, 16] (Table **S10**). We have also calculated the accuracy score of CLnc-Pred on various crops in comparison to PLEKv2 pl. It showed excellent results (Fig. **7B**). These findings indicate that our model can serve as a reliable tool for genetic research and bioinformatics applications, as it facilitates more accurate identification of lncRNAs. Our study has some limitations. First, it performs only binary classification, as it only classifies lncRNA and cRNA. The dataset used was derived from publicly available repositories, which may contain biases in labeling. Additionally, our model performs well on known lncRNA sequences, but it needs to be generalized for novel lncRNA sequences. Future efforts will focus on expanding the dataset by including underrepresented crops like barley and millet to enhance the tool’s generalizability. Furthermore, adding other biological characteristics, such as secondary structure and evolutionary conservation, would improve the classification's biological relevance and prediction accuracy. Moreover, a web-based interface will be created to provide real-time analysis and user accessibility, enabling researchers to effectively categorize lncRNAs and investigate their possible functions [63]. All things considered, our work offers a very precise, effective, and comprehensible methodology for lncRNA prediction, advancing computational biology and genomics research.

## CONCLUSION

CLnc-Pred demonstrates promising performance in distinguishing lncRNAs from cRNAs in crop transcriptomes. Future improvements may include expanding training datasets, integrating additional features, and applying advanced deep-learning approaches to enhance prediction accuracy.

## Figures and Tables

**Table 1:** Training, validation, and test sets for model development.

-	**Training Set**	**Validation Set**	**Test Set**
**lncRNA sequences** **(Positive class)**	35,200	4400	4400
**cRNA sequences** **(Negative class)**	35,200	4400	4400
**Total sequences**	70,400	8800	8800

**Table 2:** Grid search hyperparameters of the best XGBoost model.

**Hyperparameters**	**Description**
Subsample (𝑟 = 0.8)	Fraction of samples used per tree
reg_lambda (**𝜆** = 1)	L2 regularization term
reg_alpha (𝛼 = 2)	L1 regularization term
n_estimators (𝑡 = 200)	Number of boosting rounds 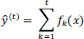
max_depth (*d* = 4)	Maximum depth of trees
learning_rate (𝝶 = 0.1)	Step size shrinkage for boosting Shrinkage factor to update predictions: 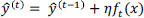
Gamma (γ = 1)	Minimum loss reduction required for further split: Split if 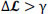
colsample_bytree (𝑐 = 0.8)	Fraction of features used per tree
eval_metric	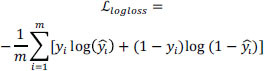 Where: 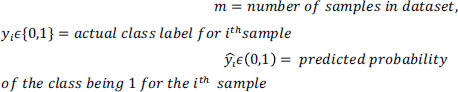

## Data Availability

The authors confirm that the data supporting the findings of this research are available on Zenodo. The datasets (FASTA files) can be accessed *via* the following link: https://zenodo.org/records/16902404?token=eyJhbGciOiJIUzUxMiJ9.eyJpZCI6ImUzOGJiNTRhLWQzY2UtNGM4Zi04Y2ZhLTczMTlkZDA2Y2M4NyIsImRhdGEiOnt9LCJyYW5kb20iOiIwMjU3NTU5NWQ5ZDM4ZTQyNDRiYmQzYWE2NzQ3MTEzOSJ9.QnEMjNfeSaGhw7wjdaC51ni47Z0ePcbqg_RkTt08gXk2fHLxSYEzBfORo3YFsfvvGrOpOHnVtAG49gmY1JqcpA

## LIST OF ABBREVIATIONS

CNCI: Coding-Non-Coding Index
CPAT: Coding-Potential Assessment Tool
CPC2: Coding Potential Calculator 2
mRNA: Coding RNA
ncRNA: Non-coding RNA
ORF: Open Reading Frame
SHAP: SHapley Additive exPlanations

## DECLARATIONOF GENERATIVE AI AND AI-ASSISTED TECHNOLOGIES IN THE WRITING PROCESS

During the preparation of this work, the author(s) used OpenAI’s ChatGPT (https://chat.openai.com) to improve the content. After using this tool/service, the author(s) reviewed and edited the content as needed and take(s) full responsibility for the content of the publication. ChatGPT was used solely for language refinement; all scientific content is original.
