# Supplementary material for: CLnc-Pred: A Machine Learning Approach to Predict Long Non-Coding RNAs in Crops
Source: Curr Genomics. 2026 Mar 19;26(6):726–39. doi: 10.2174/0113892029416615260116163601 (PMC13154242; doi:10.2174/0113892029416615260116163601)
Supplement: Supplementary file 1 [file CG-26-6-726_SD1.pdf]

Supplementary Material

CLnc-Pred: A Machine Learning Approach to Predict Long Non-Coding RNAs in Crops

Bhavesh Kumar Choubisa[1], Anu Sharma[1,*], Nitesh Kumar Sharma[1,2], Mohammad Samir Farooqi[1], Dwijesh Chandra Mishra[1], K.K. Chaturvedi[1], S.B. Lal[1], Alka Arora[1] and Girish Kumar Jha[1]

[1]ICAR-Indian Agricultural Statistics Research Institute, PUSA, New Delhi, 110012, India; [2]Dr. Rajendra Prasad Central Agricultural University, Pusa, Samastipur – 848125, Bihar, India

Table S1. Friedman and Nemenyi Post-hoc Test Results for Model Performance Comparison.

Metric	Friedman χ^2	p-value	RF	XGBoost	CNN	ANN	DNN
Accuracy	93.603	<0.0001	ade	ade	bc	bc	bc
Precision	58.000	<0.0001	ce	abde	c	c	bc
Recall	73.947	<0.0001	acde	abd	bce	bc	ab
F1-score	94.853	<0.0001	ade	ade	bc	bc	bc
AUC	105.413	<0.0001	ade	ade	bce	bc	abc

Table S2. Class-wise Performance Metrics (Mean ± SD) of XGBoost Model for cRNA and lncRNA.

Class	Precision	Recall	F1-score
cRNA	0.967 ± 0.003 (SE=0.000)	0.937 ± 0.003 (SE=0.001)	0.952 ± 0.002 (SE=0.000)
lncRNA	0.939 ± 0.003 (SE=0.001)	0.968 ± 0.003 (SE=0.000)	0.953 ± 0.002 (SE=0.000)

Table S3. Statistical Analysis of Class-wise Performance Metrics.

Metric	Paired t-test p	Wilcoxon p
Precision	3.58×10^{-27}	1.86×10^{-9}
Recall	5.07×10^{-28}	1.86×10^{-9}
F1	2.96×10^{-10}	2.35×10^{-6}

Table S4. XGBoost performance at different CD-HIT thresholds.

Metrics	CDHit_0.80	CDHit_0.85	CDHit_0.90	CDHit_0.95	CDHit_1.0
**Accuracy**	0.957 ± 0.002	0.951 ± 0.001	0.951 ± 0.001	0.951 ± 0.001	0.951 ± 0.001
**Precision**	0.944 ± 0.003	0.933 ± 0.002	0.932 ± 0.002	0.932 ± 0.002	0.932 ± 0.003
**Recall**	0.973 ± 0.002	0.980 ± 0.001	0.980 ± 0.001	0.981 ± 0.002	0.980 ± 0.002
**F1**	0.958 ± 0.002	0.956 ± 0.001	0.955 ± 0.001	0.956 ± 0.001	0.955 ± 0.001
**AUC**	0.994 ± 0.000	0.989 ± 0.000	0.989 ± 0.001	0.989 ± 0.000	0.989 ± 0.000

Table S5. XGBoost performance at different dataset ratios.

Ratio	Accuracy (Weighted Mean ± SD)	Precision (Weighted Mean ± SD)	Recall (Weighted Mean ± SD)	F1-Score (Weighted Mean ± SD)	AUC (Weighted Mean ± SD)
1:1	0.950 ± 0.002	0.912 ± 0.003	0.996 ± 0.001	0.953 ± 0.002	0.993 ± 0.000
1:2	0.952 ± 0.002	0.928 ± 0.005	0.927 ± 0.005	0.927 ± 0.003	0.993 ± 0.000
1:3	0.956 ± 0.002	0.949 ± 0.004	0.873 ± 0.007	0.909 ± 0.003	0.993 ± 0.000
1:5	0.965 ± 0.002	0.946 ± 0.008	0.841 ± 0.010	0.890 ± 0.005	0.993 ± 0.000
1:10	0.978 ± 0.002	0.943 ± 0.010	0.804 ± 0.015	0.868 ± 0.009	0.993 ± 0.001

Table S6. Dunn's Post Hoc Analysis of Model Accuracy Across Different lncRNA: cRNA Dataset Ratios.

Ratio	1:1	1:2	1:3	1:5	1:10
1:1	1	1	0.00010343	8.15E-12	8.11E-22
1:2	1	1	0.00697359	8.29E-09	1.21E-17
1:3	0.00010343	0.00697359	1	0.05982374	5.85E-07
1:5	8.15E-12	8.29E-09	0.05982374	1	0.07485072
1:10	8.11E-22	1.21E-17	5.85E-07	0.07485072	1

Table S6 presents the results of Dunn's post hoc test performed after Kruskal–Wallis analysis to assess pairwise differences in model accuracy across various lncRNA:cRNA dataset ratios. The table reports *p*-values for each comparison, where bold values ($p < 0.05$) indicate statistically significant differences between ratios. Significant differences were primarily observed between the balanced dataset (1:1) and highly imbalanced datasets (e.g., 1:10), demonstrating that class imbalance significantly affects model accuracy. In contrast, moderate ratios (e.g., 1:3 vs 1:5) showed non-significant differences, suggesting stable performance once a relatively balanced dataset is achieved.

Table S7. Dunn's Post Hoc Analysis of Model Precision Across Different lncRNA: cRNA Dataset Ratios.

Ratio	1:1	1:2	1:3	1:5	1:10
**1:1**	1	**0.02878253**	**2.41E-17**	**5.89E-14**	**1.89E-11**
**1:2**	**0.02878253**	1	**8.62E-08**	**1.39E-05**	**0.00048636**
**1:3**	**2.41E-17**	**8.62E-08**	1	1	0.90308023
**1:5**	**5.89E-14**	**1.39E-05**	1	1	1
**1:10**	**1.89E-11**	**0.00048636**	0.90308023	1	1

Table S7 shows that significant differences in precision were observed between the balanced (1:1) and more imbalanced datasets (1:3, 1:5, 1:10), indicating that class imbalance affects the precision of the model's predictions. However, comparisons among moderately or highly imbalanced ratios (e.g., 1:3 vs 1:5, 1:5 vs 1:10) were not significant, suggesting that once imbalance increases beyond a certain level, its effect on precision stabilizes.

Table S8. Dunn's Post Hoc Analysis of Model Recall Across Different lncRNA: cRNA Dataset Ratios.

Ratio	1:1	1:10	1:2	1:3	1:5
**1:1**	1	**1.49E-25**	0.07486717	**8.57E-07**	**8.29E-15**
**1:10**	**1.49E-25**	1	**1.34E-14**	**1.10E-06**	0.08924024
**1:2**	0.07486717	**1.34E-14**	1	0.07355075	**7.64E-07**
**1:3**	**8.57E-07**	**1.10E-06**	0.07355075	1	0.07035008
**1:5**	**8.29E-15**	0.08924024	**7.64E-07**	0.07035008	1

Table S8 illustrates that recall varied significantly between the balanced dataset (1:1) and more imbalanced datasets (e.g., 1:10, 1:3, 1:5), as shown by the highly significant p-values. These results indicate that class imbalance substantially reduces the model's ability to correctly identify lncRNA sequences. In contrast, comparisons among moderately imbalanced ratios (e.g., 1:2 vs 1:3 or 1:3 vs 1:5) showed no significant differences, suggesting recall stabilizes once the dataset becomes sufficiently imbalanced.

Table S9. Dunn's Post Hoc Analysis of Model F1-Score Across Different lncRNA: cRNA Dataset Ratios.

Ratio	1:1	1:10	1:2	1:3	1:5
**1:1**	1	**1.23E-25**	0.07486611	**8.86E-07**	**9.13E-15**
**1:10**	**1.23E-25**	1	**1.16E-14**	**9.61E-07**	0.08177035
**1:2**	0.07486611	**1.16E-14**	1	0.07486611	**8.16E-07**
**1:3**	**8.86E-07**	**9.61E-07**	0.07486611	1	0.07161400
**1:5**	**9.13E-15**	0.08177035	**8.16E-07**	0.07161400	1

Table S9 demonstrates that the F1-score differed significantly between the balanced dataset (1:1) and more imbalanced datasets (e.g., 1:10, 1:3, 1:5), indicating that class imbalance reduces the model's overall balance between precision and recall. However, comparisons among moderately imbalanced datasets (e.g., 1:2 vs 1:3, 1:3 vs 1:5) showed no significant differences, suggesting that once the dataset becomes sufficiently skewed, further imbalance does not substantially affect the F1-score.

Table S10. Performance comparison of CLnc-Pred with existing tools.

**Tools**	**Accuracy**	**Precision**	**Recall (Sensitivity)**	**Specificity**	**F_1.Score**	**Average time taken per seq.**
CPC2	90.20%	90.09%	90.27%	90.14%	90.18%	10-12 ms
PLEKv2 pl	83.06%	81.31%	86.30%	79.75%	83.73%	240-300 ms
CLnc-Pred	95.87%	94.43%	97.41%	94.37%	95.92%	7-10 ms

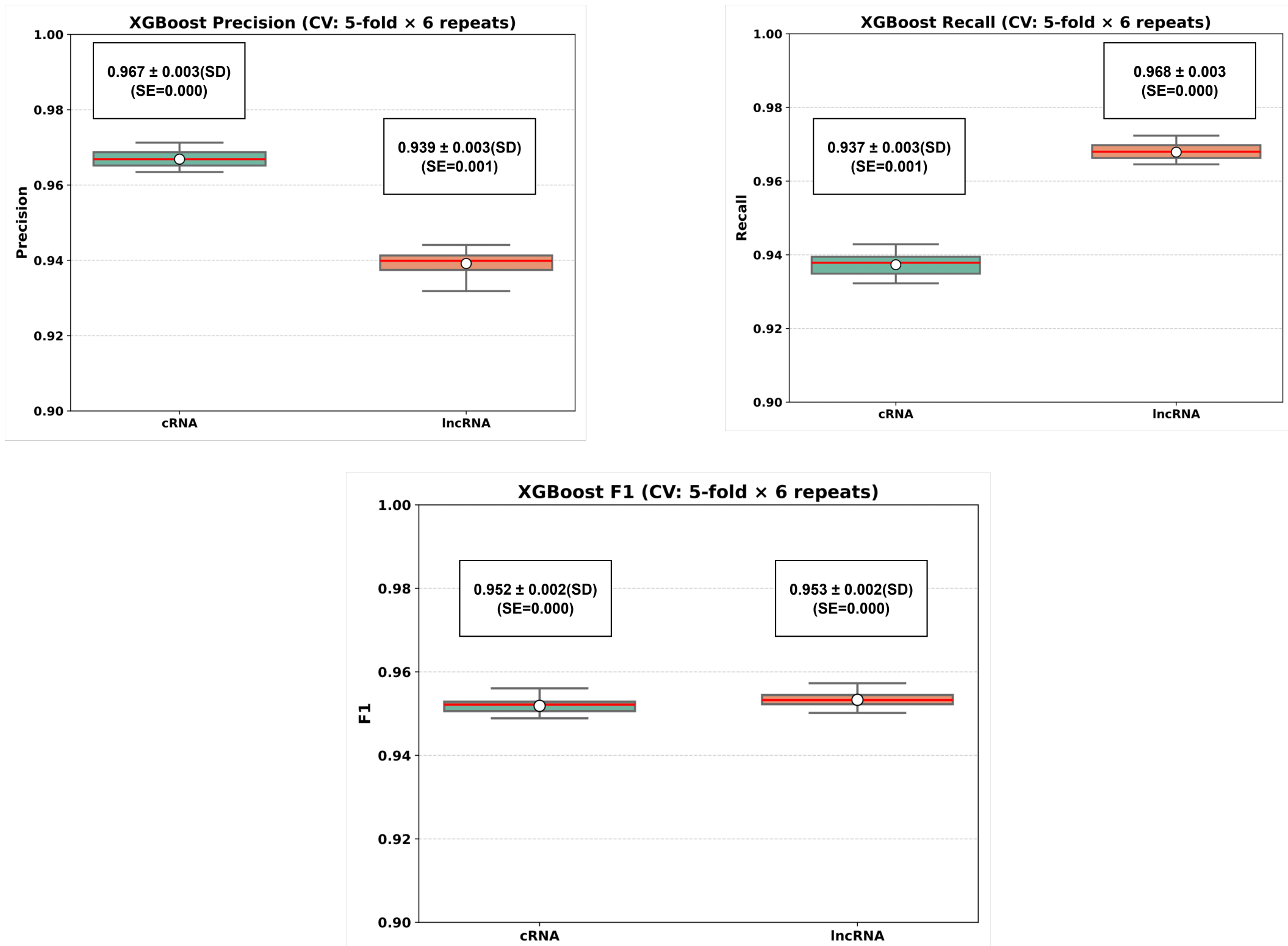

Fig. (S1). Class-wise classification performance of XGBoost.

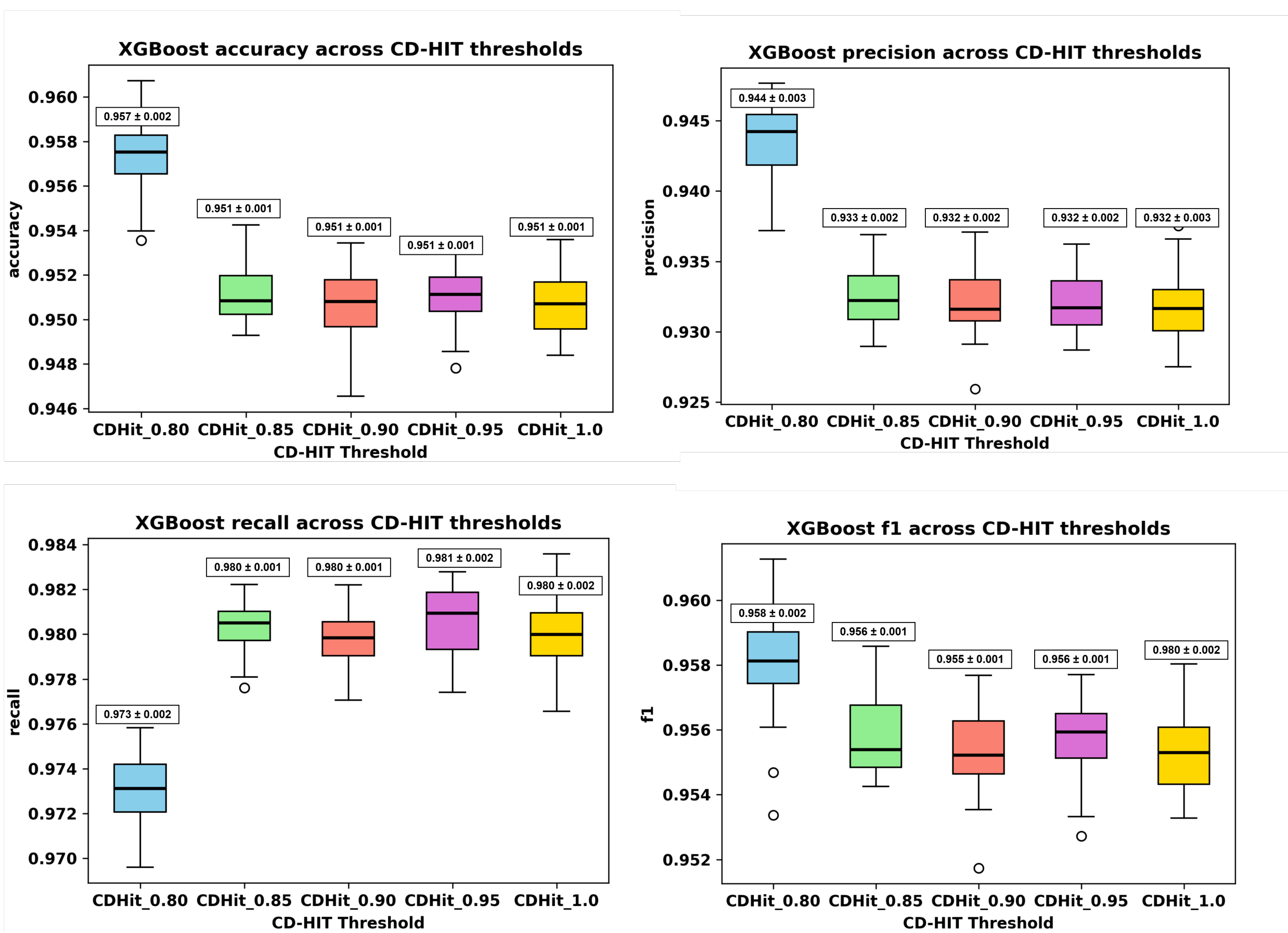

Fig. (S2). XGBoost Performance (Mean± SD) at Different CD-HIT Thresholds.

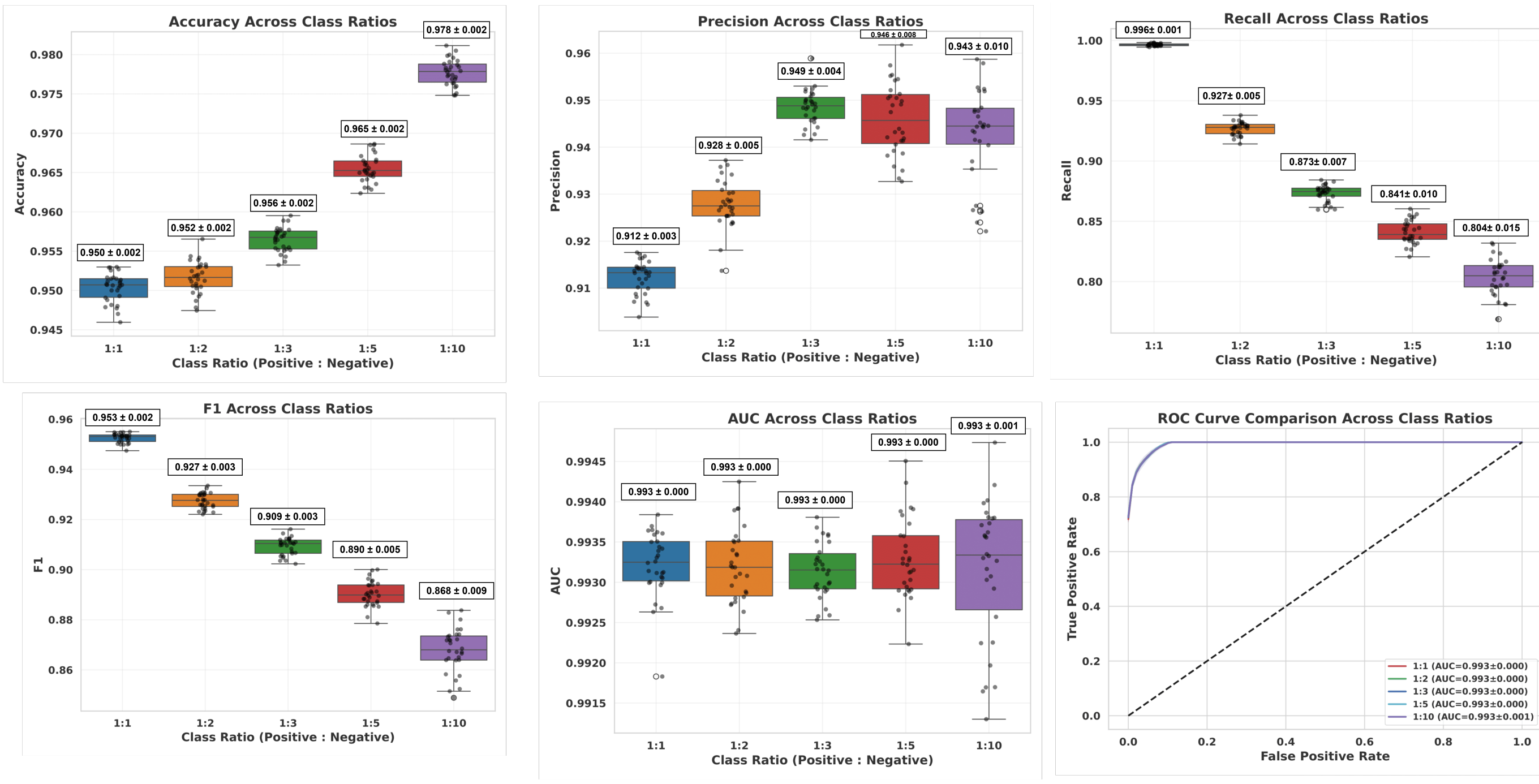

Fig. (S3). Model performance across different class ratios (lncRNA: cRNA).
